# Impact of single nucleotide polymorphisms (SNPs) in antioxidant-enzyme genes on the concentrations of folate, homocysteine and glutathione in plasma from healthy subjects after folic acid supplementation – a randomized controlled crossover trial

**DOI:** 10.1186/s12263-024-00761-6

**Published:** 2025-01-21

**Authors:** Mohammad Azam Mansoor, Tonje Holte Stea, Audun Slettan, Erandie Perera, Ridmi Maddumage, Darshana Kottahachchi, Dhikra Saleem Ali, Rona Cabo, Rune Blomhoff

**Affiliations:** 1 Department of Natural Sciences, University of Agder (UiA), Kristiansand, 4604 Norway; 2 Department of Health and Nursing Sciences, University of Agder (UiA), Kristiansand, Norway; 3 Department of Medical Laboratory Sciences, General Sir John Kotelawala Defence University (KDU), Colombo, Sri Lanka; 4 Department of Nutrition, University of Oslo (UiO), Oslo, Norway; 5 Department of Clinical Service, Division of Cancer Medicine, Oslo University Hospital, Oslo, Norway

**Keywords:** Single nucleotide polymorphisms (SNPs), Allele, Antioxidant-enzyme genes, Antioxidant enzymes, Antioxidant defense system, Free radicals, S-folate, P-tHcy, P-tGSH, Folic acid

## Abstract

**Background:**

One-carbon metabolism links folate and methionine metabolism and this is essential for nucleotide synthesis in the cells. Alterations in one-carbon metabolism are associated with cardiovascular disease (CVD), type 2 diabetes and cancer. Our aim was to investigate whether SNPs in antioxidant-enzyme genes impact the concentrations of folate in serum (s-folate), plasma total homocysteine (p-tHcy) and total glutathione in plasma (p-tGSH) in healthy subjects after supplementation with folic acid.

**Methods:**

In a randomized, double blind, crossover study, healthy subjects received 0.8 mg folic acid per day or a placebo for two weeks. Twenty-four male, and sixty-seven female subjects participated in this study. Participants were aged 36.4 ± 14.8 years (mean ± SD). We studied SNPs in six genes by PCR methods. The concentrations of s-folate, p-tHcy and p-tGSH were measured in fasting samples with Cobas and an HPLC-fluorescence method. Student T-tests and ANOVA were used for the statistical calculations.

**Main findings:**

The subjects with SNP (rs4880) in superoxide dismutase (SOD2) gene (CC) allele had higher concentrations of s-folate and lower concentrations of p-tHcy than subjects with (CT + TT) alleles, (*p* = 0.014 and *p* = 0.012). Contrary to SOD2 (CC) allele, the subjects with SNP (rs1001179) catalase (CAT) CC allele had lower concentrations of s-folate (*p* = 0.029), higher concentrations of p-tGSH (0.017) and higher concentrations of p-tHcy before and after folic acid supplementations (*p* = 0.015, *p* = 0.017) than the subjects with (CT + TT) allele. Glutathione transferase (theta)1 (GST-T1) genotype was associated with higher concentrations of s-folate than GST-T0 before (*p* = 0.025) and after folic acid supplementation (*p* = 0.047). SNP (rs1050450) in glutathione peroxidase (GPX1) had also impact on the concentrations of p-tGSH (*p* = 0.011) in healthy subjects.

**Conclusion:**

SNPs in *SOD2 (rs4880), CAT (rs1001179), and GST1* impact the concentrations of s-folate, and p-tHcy in healthy subjects before and after folic acid supplementation. Our findings suggest that SNPs in antioxidant-genes have a role in health and disease by impacting the concentrations of s-folate, p-tHcy and p-tGSH.

## Introduction

One-carbon metabolism integrates folate- and methionine-homocysteine pathways, and this is also pivotal for the synthesis of nucleotides in the cells. Furthermore, one-carbon metabolism provides methyl-groups to methyl-group acceptors for the regulation of epigenetics that contributes to maintaining biochemical homeostasis in the cells [1, 2]. Several studies have reported that impaired one-carbon metabolism is associated with increased prevalence of CVD, type 2 diabetes and cancer [1, 3, 4].

Previously, we have reported that folic acid supplementation increases the concentration of s-folate in healthy subjects [5]. The main form of folate in the blood serum is 5-methyltetrahydrofolate (5-MTHF) which provides methyl-group to methyl-group acceptor in the folate methioine-homocysteine pathway in the cells. In this pathway, vitamin B_12_-accepts a methyl-group and remethylates homocysteine into methionine in the cells. Homocysteine formed in the cells is converted into cysteine by the activity of the enzymes cystathionine-β-synthase and cystathionine lyase, which require pyridoxal-5-phosphate (5-PLP) (vitamin B_6_) as a coenzyme [6]. The bioavailability of cysteine in the blood depends on the catabolism of proteins and release of cysteine from the small intestine into the blood, and from methionine metabolism in the cells. Cysteine produced during methionine metabolism, or obtained through the diet, is used for the synthesis of glutathione (GSH) which is the most important and an essential antioxidant in the body [7–9].

Deficiency of folate, vitamin B_12_ and vitamin B_6_ will cause an increase in the concentration of p-tHcy and may impact the concentration of p-tCys and p-tGSH [6]. Since GSH is the major thiol antioxidant, a low concentration of p-tGSH may reflect deficiency of cysteine, or an increased production and/or higher concentrations of free radicals or oxidants in the blood. An increased concentration of free radicals, such as superoxide (O_2_^**•¯**^), hydroxyl radical (HO^•^), and oxidant hydrogen peroxide (H_2_O_2_), lead to oxidization of biomolecules in the blood [10–16]. A higher concentration of oxidized species compared to antioxidants in the blood results in increased oxidative stress and this is associated with increased risk of developing CVD, cancer, type 2 diabetes and neurological dysfunction [17–23]. Even though high concentrations of O_2_^**•¯**^ and H_2_O_2_ are toxic to the cells, at normal concentrations these chemical species may play an important role in signaling pathways within the cells [24, 25].

Several studies have reported that low concentrations of s-folate, and p-tGSH and high concentrations of p-tHcy are associated with CVD and type 2 diabetes, and these are responsible for increased morbidity in elderly population [4, 26–33].

The enzymes SOD2, CAT, GPX1, glutathione reductase (GSR), glutathione transferase, isoforms GST-theta (GST-T1), and GST-mu (GST-M1) make up the biochemical antioxidant defense system in the body. Folate or vitamin B9 is an antioxidant, whilst homocysteine is an oxidant; we therefore intended to study the impact of SNPs in genes coding antioxidant enzymes on the concentrations of s-folate, p-tHcy and p-tGSH. GSH is an essential antioxidant used by the enzymes GPX and GST to neutralize oxidants and remove electrophiles (xenobiotics) from the body [34–41] (Table 1). SNPs in genes coding antioxidant-enzyme may affect their mRNA status, reduce or increase the enzyme activity and possibly impact their antioxidant ability. Therefore, it has been suggested that SNPs in antioxidant-enzyme genes may be associated with CVD, type 2 diabetes, cancer and neurodegenerative diseases [34–41].

**Table 1:** SNPs and the primers used in PCR methods

Gene and enzyme function	SNPs	Primers, method
Superoxide dismutase 2 *(SOD2)* (Activity in mitochondria) 2O_2_^−.^ + 2H^+^ + SOD2 (Mn) → H_2_O_2_ + O_2_	SNP ID: rs4880 (A → G). This SNP converts amino acid valine to alanine at the amino acid position 16 of the protein	Thermofisher TaqMan SNPassay ID C_8709053_10
Catalase *(CAT)* H_2_O_2_ + CAT → H_2_O + ½ O_2_	SNP ID: rs1001179, (− 262 C → T) (promotor region, 262 bp before the start site of transcription	F: 5´-TAA GAG CTG AGA AAG CAT AGC T-3´ R: 5´-AGA GCC TCG CCC CGC CGG ACC G-3´
Glutathione peroxidase1 *(GPX1)* H_2_O_2_ or LOOH + 2GSH + GPX → H_2_O or LOH + H_2_O + GS-SG	SNP ID: rs1050450 (C → T). This SNP converts proline to leucine in the position 198 amino acid in the protein. Wild type homozygote CC, 198 Pro/Pro, Heterozygote CT 198, Pro/Leu and homozygote variant TT, 198 Leu/Leu	F: 5´-TCC AGA CCA TTG ACA TGG AG-3´ R: 5´-ACT GGG ATC AAC AGG ACC AG-3´
Glutathione reductase (GSR) GS-GS + NADPH + H^+^ + GSR → 2 GSH + NADP^+^	SNP ID:rs2978663 Intron 3 (GSRint3) (A → T)	F: 5´TAC CGG GTT CAC GCC ATT CT-3´ R: 5´-GTT AAA AGC CTC TGT GCC ACT CAG-3´
Glutathione S-transferase (GST) Xenobiotic → Hydroxylation → Xenobiotic-OH + GSH + GST → Xenobiotic-SG → Xenobiotic-SG excreted	GST Mu1 (GST-M1 and GST-M0) GST Theta1 (GST-T1) and GST-T0) GST-M0 and GST-T0 (Genes are absent)	F: 5´-GAA CTC CCT GAA AAG CTA AAG C-3´ R: 5´-GTT GGG CTC AAA TAT ACG GTG G-3´ F: 5´-TTC CTT ACT GGT CCT CAC ATC TC-3 R: 5´-TCA CCG GAT CAT GGC CAG CA-3´ β-globulin-F: 5´-GAA GAG CCA AGG ACA GGT AC-3´ β-globulin-R: 5´-CAA CTT CAT CCA CGT TCA CC-3´

GS-SG is an oxidized form of GSH, *LOOH* Lipid peroxide, *NADP* Nicotinamide adenine dinucleotide phosphate, Xenobiotic-SG is xenobiotic conjugated with GSH

Our aim was to investigate the impact of SNPs in genes coding antioxidant-enzymes SOD2, CAT, GPX, GSR, and GST-T1 and GST-M1 on the concentrations of s-folate, p-tHcy and p-tGSH in healthy subjects following folic acid supplementation. To our knowledge, it has not been reported whether SNPs in genes coding antioxidant-enzymes impact the concentrations of s-folate, p-tHcy and p-tGSH following folic acid supplementation.

## Materials and methods

### Study design and the participants

A total of 103 healthy employees and students from the University of Agder, Norway, agreed to participate in the randomized, double blind, crossover study. Self-reported health status was provided by a questionnaire that was filled out by all the participants. Pregnant women, and individuals reporting use of vitamin supplements or drugs, and individuals suffering from cardiovascular disease, cancer and type 2 diabetes were not included in this study. During the first two weeks of the study, 52 participants received 0.8 mg folic acid once daily and 51 participants received a placebo. After a two weeks wash-out period, individuals who had initially received a placebo received folic acid for another two weeks and vice versa. During the study period twelve participants dropped out, with a total of 91 individuals aged 36.4 ± 14.8 years old (mean ± SD) with BMI of 24.6 ± 3.7 (mean ± SD) completing the study. We used the data on folic acid supplementation (*n* = 91) and placebo (*n* = 45) in this study. A flow chart describing the recruitment and follow-up of participants have been published previously [5, 42].

The present study was performed in line with the guidelines established by the Declaration of Helsinki. The Regional Ethics Committee for Medical Research (REK), and the Norwegian Data Inspectorate approved the study protocol. The trial number of this study is REK. S-06278a.

### Blood samples

Fasting blood samples were collected in the morning (between 7 and 9 am) before and after supplementation of folic acid and placebo (day 1, 15, 30 and 45). All blood samples were placed on the ice in the dark before being centrifuged at 4 °C for 15 min at 1500 rpm within 30 min. Serum and plasma samples were stored at − 80 °C before analysis.

### Measurement of p-tHcy, p-GSH and s-folate

Heparin plasma was used for the measurement of p-tHcy and p-tGSH concentrations using a HPLC method with fluorescence detection [43]. The HPLC method has coefficient of variation (CV%) < 8% for all aminothiols. Serum was used for the measurement of folate using a Cobas 6000 at the Laboratory of Medical Biochemistry, Sørlandet Hospital, Arendal. The analytical method for the measurement of serum folate has a CV 5%. The concentrations of s-folate and p-tHcy provided in the present manuscript have been reported previously [5].

### DNA extraction and PCR assay

Genomic DNA was purified from 100 µl whole blood using DNeasy Blood & Tissue Kit (Qiagen inc.) and QIAcube automated DNA extraction system (Qiagen inc.) The protocol of the reagent’s provider was followed. The DNA was eluted in 200 µl AE buffer, and the DNA concentration and its purity (A_260_/A_280_) was determined on a NanoDrop 1000 Spectrophotometer (Thermo Scientific Inc.) following the producer’s protocol. All samples were genotyped for SNPs: GSR int3 (rs2978663), CAT −262 (rs1001179), SOD2 Val16Ala (rs4880) using AmpliTaq Genotyping Master Mix (Applied Biosystems) and TaqMan SNP Genotyping Assay (Applied Biosystems) for each respective SNP. Each genotyping reaction mix consisted of 10 µl 2 × AmpliTaq Genotyping Master Mix, 1 µl 20 × SNP Genotyping Assay, 2 µl DNA (10 – 15 ng DNA) and 7 µl H_2_0. The analysis was performed at a StepOne Plus Real-Time PCR system (Thermo Fisher Inc.) with the following temperature profile: 60 °C for 30 s; 95 °C for 10 min; 45 cycles of 95 °C for 15 s followed by 60 °C for 30 s. Fluorescence detection was done after each cycle. The genotyping software of the StepOne Plus machine analyzed the results and categorized the genotype of each sample. The studied polymorphisms of the GSTT1 and GSTM1 genes are of the form null alleles due to deletion of the genes and were analyzed by PCR with subsequent agarose gel electrophoresis. Protocol and PCR primers for the genes as well as internal control was prepared according to methods as reported previously [44]. Briefly: Primers for genes GSTT1 and GSTM1 together with primers for internal control gene *HBB* (β globulin) were used in a 25 µl multiplex PCR reaction containing 1,5 mM MgCl_2_, 0,3 mM dNTP, 0,4 µM of each primer, 1,5 U Amplitaq -Gold DNA polymerase (Applied Biosystems) with the corresponding 1 × PCR buffer (Applied Biosystems) and 0,1 µg DNA. Temperature profile was 95° C for 5 min. followed by 35 cycles at 95° C for 1 min, 65° C for 1 min and 72° C for 1 min. and a final extension at 72° C for 5 min. 10 µl PCR products were analyzed by 2% agarose gel electrophoresis. *GST-T1* positive fragment has a length of 459 bp, *GST-M1* positive fragment is 209 bp and the length of the internal control gene, HBB, is 268 bp. The SNP (rs1050450) in the GPX1 gene was analyzed according to previously published report (Tang NP 2008). Briefly, PCR primers with a final concentration 0,3 µM, 1,5 mM MgCl_2_, 0,2 mM dNTP, 1,5 U Amplitaq Gold DNA polymerase (Applied Biosystems) with the corresponding 1 × PCR buffer (Applied Biosystems) and 0,2 µg DNA were used in the assay. PCR temperature profile was 95° C for 8 m, 35 cycles at 94° C for 30 s, 59° C for 30 s and 72° C for 30 s followed by a final incubation at 72° C for 7 min. 5 µl PCR product was cut with restriction enzyme ApaI (New England BioLabs inc.) and analyzed by 2% agarose gel electrophoresis. CC genotype will result in two fragments of 170 bp and 52 bp. The restriction enzyme will not cut the fragments from a person with the TT genotype and therefore give a fragment of 222 bp. Heterozygous genotype will give all three fragments of 222 bp, 170 bp and 52 bp (Table 1) [45–47].

### Statistical methods

The distribution of aminothiols in plasma is not normal, therefore, we converted the concentration values of s-folate, p-tHcy and p-GSH into log values [26]. We used student T-test for the comparison of data between two groups and student paired T-test to study the change from the baseline to after supplementation with folic acid or placebo. We used ANOVA and Bonferroni post-hoc test for multiple comparisons in this study. Double sided, *p*-value < 0.05 was used to show a significant difference in the concentration or change in the concentrations of s-folate, p-tHcy and GSH. We used IBM SPSS Statistics 25 for all statistical analyses.

## Results

We discovered that the distribution of allele frequencies was accordance to the Hardy Weinbergs equilibrium (Table 2).

**Table 2:** Allele frequency of genes investigated in the present study

Gene	Allele frequencyHWE Test	(*p*-value)
SOD T > C, C47T (Ala 16 Val)		
T allele	0.41	0.51
G allele	0.59	
Catalase C > T, (-C262T) (change in promotor region)		
C Allele	0.8	0.07
T allele	0.2	
Glutathione peroxidase, GPX C > T (C602T, Pro198Leu)		
T allele	0.36	0.349
C Allele	0.64	
Glutathione reductase, GSR A > G (Intron 3, GSRint A > G)		
A allele	0.36	
G allele	0.64	
Glutathione transferase (GST)-T (T1 present, GST-T0, absent)		
GST-T1	0.89	
GST-T0	0.11	
Glutathione transferase (GST)-M, (GST-M1, present, GST-M0, absent)		
GST-M1	0.46	
GST-M0	0.54	

*HWE test *Hardy–Weinberg test,*

As reported previously, the concentrations of s-folate increased from 17.2 ± 6.3 nmol/L to 40.1 ± 30.7 nmol/L (mean ± SD) (*p* < 0.001), and the concentrations of p-tHcy decreased from 8.4 ± 3.5 µmol/L to 7.8 ± 3.0 µmol/L (mean ± SD), (*p* < 0.001) and (*p* < 0.001) following folic acid supplementation. However, the concentrations of GSH did not change significantly (from 4.2 ± 1.7 to 4.4 ± 1.8 µmol/L) (mean ± SD) (*p* > 0.05) in healthy subjects after supplementation with folic acid (Table 3).

**Table 3:** Changes in the concentrations of s-folate, p-tHcy and glutathione after short term intervention with folic acid in healthy subjects (dose) for X days/weeks

	**Baseline**			**After supplementation**			**Within-group mean changes after supplementation**			
	**Intervention group** **(*n*=87)**	**Control group** **(*n*=45)**	***p*** -value*	**Intervention group**	**Control group** **(*n*=45)**	***p*** -value*	**Intervention group**	**Control group**	**β-** ** value (95% CI)**	***p*** **-value** ^#^
s-folate	17.2 (15.8, 18.5)	16.4 (14.7, 18.1)	0.527	40.3 (33.7, 47.0)	17.5 (15.7, 19.4)	**<0.001**	23.1 (16.6, 29.7)	1.11 (0.1, 2.1)	**−0.57 (−0.36, −0.23)**	**<0.001**
p-tHcy	8.5 (7.7, 9.2)	8.8 (7.6, 10.1)	0.535	7.8 (7.1, 8.4)	8.5 (7.6, 9.4)	0.062	−0.7 (−1.0, −0.4)	−0.3 (−1.0, 0.3)	**0.12 (0.01, 0.06)**	**0.021**
p-GSH	4.2 (3.9, 4.6)	4.4 (4.0, 4.8)	0.287	4.4 (4.1, 4.8)	5.0 (4.4, 5.7)	0.070	0.2 (−0.1, 0.5)	0.6 (0.2, 1.1)	0.09 (−0.01, 0.07)	0.131

To obtain normality, s-folate, p-Hcy, and p-GSH, concentrations were log_e_ transformed before comparison

*Significant difference (*p* < 0.05) between the intervention group and the control group, analyzed using an unpaired *t* test

^#^Significant difference (*p* < 0.05) between mean scores at baseline and after folic acid supplementation in each variable (of each group) adjusted for baseline scores, using linear regression mod

Subjects with SNP (rs4880) in SOD*2* gene (*CC)* allele had higher concentrations of s-folate (*p* = 0.014) and lower concentrations of p-tHcy before and after supplementation with folic acid (*p* = 0.012 and *p* = 0.004) than in the subjects with (*CT* + *TT)* alleles (Table 4).

**Table 4:** Concentration of s-folate, p-tHcy and p-GSH before and after folic acid supplementation according to gene polymorphisms

		s-folate (nmol/L)			p-tHcy (μmol/L)			p-GSH (μmol/L)		
Gene	Suppl	Before suppl	After suppl	*p-value*	Before suppl	After suppl	*p-value*	Before suppl	After suppl	*p-value*
*SOD2 C>T*										
CC (*n*=30)	FA	**18.5±5.4 (1)**	41.8±23.2	*<0.001*	**7.6±2.0 (2)**	**7.1±1.3 (3)**	*0.003*	4.2±1.4	4.4±1.8	*0.189*
CC (*n*=14)	Placebo	18.3±5.3	18.9±4.5	*0.29*	7.4±1.3	7.5±1.2	*0.85*	4.3±1.1	4.6±1.3	*0.270*
	*p-value*	*0.805*	*<0.001*		*0.601*	*0.102*		*0.640*	*0.431*	
CT+TT (*n*=61)	FA	16.6±6.6	39.4±33.6	*<0.001*	8.8±4.0	8.1±3.5	*0.003*	4.3±1.8	4.4±1.8	*0.277*
CT+TT (*n*=32)	Placebo	15.6±5.6	16.9±6.8	*0.034*	9.3±4.9	9.0±3.2	*0.470*	4.5±1.5	5.2±2.5	*0.030*
	*p-value*	*0.642*	*<0.001*		*0.300*	*0.073*		*0.264*	*0.135*	
*CAT C>T*										
CC (*n*=54)	FA	16.5±6.2	40.3±29.6	*<0.001*	**8.6±4.1 (5)**	**8.2±3.5 (6)**	*0.002*	**4.6±1.9 (7)**	4.5±2.0	*0.511*
CC (*n*=30)	Placebo	15.6±5.6	17.5±6.9	*0.002*	9.4±4.9	8.9±3.2	*0.521*	4.5±1.5	5.4±2.3	*0.003*
	*p-value*	*0.983*	*<0.001*		*0.896*	*0.066*		*0.977*	*0.029*	
CT+TT (*n*=37)	**FA**	**18.3±6.4 (4)**	39.9±32.7	<0.001	7.8±2.2	7.2±1.8	*0.040*	3.7±1.1	4.3±1.5	*<0.001*
CT+TT (*n*=16)	Placebo	18.0±5.4	17.6±4.8	0.46	7.6±2.2	7.8±1.6	*0.858*	4.2±1.2	4.4±1.8	*0.839*
	*p-value*	*0.983*	*<0.001*		*0.896*	*0.066*		*0.172*	*0.891*	
*GPX C>T*										
CC (*n*=35)	FA	16.1±4.8	34.3±18.3	<0.001	8.4±4.8	7.8±4.0	0.107	**3.9±1.5 (8)**	4.3±1.5	*0.020*
CC (*n*=15)	Placebo	16.8±5.3	17.1±4.1	0.28	9.0±6.7	8.5±4.4	0.435	4.0±1.2	4.6±1.7	*0.090*
	*p-value*	*0.649*	*<0.001*		*0.948*	*0.729*		*0.633*	*0.625*	
CT+TT (*n*=43)	FA	18.3±7.3	39.4±26.4	<0.001	8.5±2.5	7.7±2.3	<0.001	4.6±1.7	4.7±2.1	*0.452*
CT+TT (*n*=22)	Placebo	16.9±5.9	18.7±7.6	0.05	8.5±2.1	8.4±1.6	0.839	4.5±1.2	5.2±2.0	*0.090*
	*p-value*	*0.537*	*<0.001*		*0.713*	*0.007*		*0.650*	*0.237*	
*GSR A>G*										
AA (*n*=39)	FA	17.2±4.9	34.7±19.4	<0.001	8.6±4.6	7.8±3.9	*<0.001*	4.1±1.4	4.4±1.5	*0.083*
AA (*n*=18)	Placebo	15.6±4.7	16.7±5.1	0.08	9.4±6.3	9.0±4.0	*1.000*	4.7±1.8	5.2±2.6	*0.433*
	*p-value*	*0.364*	*<0.001*		*0.948*	*0.059*		*0.244*	*0.376*	
AG+GG (*n*=51)	FA	17.4±7.2	42.7±34.8	<0.001	8.8±5.1	8.2±3.9	*0.09*	4.4±1.9	4.5±1.9	*0.500*
AG+GG (*n*=28)	Placebo	16.9±6.1	18.1±6.9	0.05	8.3±2.5	7.7±2.1	*0.56*	4.2±1.2	4.9±1.9	*0.012*
	*p-value*	*0.99*	*p<0.001*		*0.498*	*0.173*		*0.635*	*0.141*	
*GST-T (1-0)*										
GST-T1 (*n*=74)	FA	**17.7±6.6 (9)**	**41.2±31.5 (10)**	<0.001	8.2±2.4	7.6±2.0	0.001	4.2±1.7	4.4±1.8	*0.180*
GST-T1(*n*=39)	Placebo	16.9±5.7	18.0±6.4	0.068	8.4±2.0	8.2±1.9	0.936	4.4±1.5	5.0±2.2	*0.060*
	*p-value*	*0.664*	*<0.001*		*0.642*	*0.022*		0.290	0.122	
GST-T0 (*n*=11)	FA	14.4±3.4	27.0±10.9	0.004	10.1±8.5	9.2±7.2	*0.169*	4.4±1.9	3.6±1.8	*0.213*
GST-T0 (*n*=5)	Placebo	13.4±4.5	14.4±3.8	0.138	12.1±11.7	10.4±7.0	*0.893*	4.2±0.8	5.6±2.3	*0.080*
*p-value 0.510*		*0.011*		*0.903*	*0.624*		*0.865*	*0.865*		
*GST-M (1-0)*										
GST-M1 (*n*=39)	FA	16.4±6.0	37.4±28.4	<0.001	9.1±4.5	8.1±4.0	0.001	4.3±1.8	4.4±1.5	*0.066*
GST-M1 (*n*=20)	Placebo	15.4±5.1	17.0±7.5		9.4±5.9	9.0±3.9	0.968	4.5±1.7	5.4±2.5	*0.005*
	*p-value*	*0.637*	*<0.001*		*0.612*	*0.130*		*0.644*	*0.144*	
GST-M0 (*n*=46)	FA	18.0±6.6	41.0±31.5	<0.001	7.9±2.6	7.5±2.1	0.069	4.2±1.6	4.4±2.0	*0.559*
GST-M0 (n=24)	Placebo	17.5±6.0	18.2±5.1	0.103	8.3±2.2	8.1±1.7	0.841	4.3±1.2	4.7±1.9	*0.475*
	*p-value*	*0.734*	*<0.001*		*0.313*	*0.087*		*0.344*	*0.276*	

Comparison between homozygote wild type, homozygote and heterozygote variant forms; (1), *p*=0.014; (2), *p*=0.012; (3), *p*=0.004; (4), *p*=0.029; (5), *p*=0.015; (6), *p*=0.017; (7), *p*=0.011; (8), *p*=0.028; (9), *p*=0.025; (10), *p*=(0.047

Contrary to subjects with SNP in *SOD2* gene, the subjects with SNP (rs1001179) in *CAT* gene (CC) allele had lower concentrations of s-folate (*p* = 0.029) and higher concentrations of p-tHcy before and after folic acid supplementation than in subjects with (*CT* + *TT)* alleles (*p* = 0.015, and *p* = 0.017). The subjects with (CC) allele had higher concentrations of GSH than the subjects with (*CT* + *TT)* alleles (*p* = 0.011) (Table 4).

Concentrations of s-folate, p-tHcy and p-tGSH were not significantly different in subjects with SNP (rs2978663) in *GSR gene, (AA) allele* and *(AG* + *GG),* alleles (Table 4).

The SNP (rs1050450) in *GPX1* gene (CC) allele was associated with lower concentrations of p-tGSH than individual with (*CT* + *TT*) alleles (*p* = 0.028) (Table 4).

Subjects with *GST-T1* genotype had higher concentrations of s-folate than subjects with *GST-T0* before and after folic acid supplementation (*p* = 0.025 and *p* = 0.047), whereas there was no significant difference in the concentrations of s-folate, p-tHcy and GSH in subjects with *GST-M1* and *GST-M0* genotype (Table 4).

## Discussion

The current study demonstrates that specific and relevant SNPs in antioxidant-enzyme genes have an impact on the concentration of s-folate, p-tHcy and p-tGSH in healthy subjects after folic acid supplementation.

### Impact of SNPs (rs4880) in SOD2 gene and (rs1001179) in CAT gene on the concentrations of s-folate, p-tHcy and p-tGSH

Our study shows that subjects with SNP (rs4880) in *SOD2 gene (CT* + *TT) alleles* have lower concentrations of s-folate and higher concentration of p-tHcy than subjects with *(CC) alleles.* These findings are novel and interesting considering previous reports where *SOD2 (CT* + *TT)* alleles have been associated with increased risk of CVD and type 2 diabetes, possibly due to decreased level or reduced activity of the SOD2 enzyme [48, 49]. However, it has not been reported whether (*CT* + *TT*) alleles are associated with CVD and type 2 diabetes due to their direct impact on the concentrations of s-folate and p-tHcy.

Previous studies have reported that *SOD2 (CT* + *TT)* alleles are associated with 25% reduced catalytical activity of the enzyme, which are potentially disrupting the cellular redox homeostasis [50, 51]. It has also been demonstrated that SOD2 is composed of four proteins synthesized in the cytosol, which are later transported into the mitochondria with the aid of a specific protein, a mitochondrial targeting sequence (MTS) protein. A SNP (rs4880) in *SOD2* gene causes a substitution of valine with alanine at the amino acid position 16 of MTS protein (valine to alanine). This change in the amino acid sequence in MTS protein converts MTS α-helix into MTS β-sheet form. A MTS protein containing β-sheet is much less efficient in aiding transport into mitochondria than the MTS with an α-helix [50–52]. Thus, (*CT* + *TT)* alleles may be associated with a lower concentration of *SOD2* in the mitochondria which may disturb folate homeostasis in the mitochondria and cells.

Contrary to *SOD2* genotype, subjects with SNP (rs1001179) in CAT *gene (CC*) allele had lower concentrations of s-folate and higher concentrations of p-tHcy than subjects with *(CT* + *TT)* alleles. This is a novel observation but supported by previous data showing that the subjects with *CAT* (*CC) allele* had lower concentrations of catalase enzyme in the blood than subjects with *(CC* + *CT)* alleles. Moreover, it has been reported that the *T* allele in *CAT* genotype had significantly higher transcriptional activity than *C* alleles [53, 54].

The subjects with *CAT (CC)* allele had higher concentrations of p-tGSH than the subjects with *(CT* + *TT) alleles*. The question remains whether GSH plays a compensatory antioxidant role when the CAT enzyme is found in decreased concentrations or has reduced catalytical activity in the cells. It has also been reported that the relationship between *CAT* genotype and phenotype may be modified by the dietary factors and the ethnicity of the participants in clinical studies [36].

### Impact of GST-T and GST-M genotypes on the concentration of s-folate and p-tHcy

The enzymes coded by *GST-T* and *GST-M* genes detoxify electrophiles by catalyzing the conjugation reaction between GSH and electrophiles in the cells. The xenobiotic-GS conjugates or GS-electrophile conjugates are removed from the body through the kidneys via urine [55–58]. Previously, it has been reported that *GST-T0 and GSTM-T0 genotypes* show polymorphic deletions with zero enzyme activity [55–58].

An interesting finding from the present study is that the subjects with *GST-T1* genotype had significantly higher concentrations of s-folate than the subjects with *GST-T0,* both before and after folic acid supplementation. The increase in the concentrations of s-folate in individuals with *GST-T1* was nearly two-fold after folic acid supplementation. This increase in the concentration of s-folate due to *GST-T1* genotype was nearly identical to the increase in the concentration of s-folate impacted by methylene-tetrahydrofolate reductase (MTHFR) after folic acid supplementation [5]. Our study also shows that *GST-M1* and *GST-M0* do not have any impact on the concentrations of s-folate, p-tHcy or p-tGSH.

### Impact of SNP in (rs1050450) in GPX1 gene and SNP (rs2978663) in GSR gene on the concentrations of s-folate, p-tHcy and p-tGSH

The mean concentration of p-tGSH measured in the present study was nearly identical to the concentration of p-tGSH reported in a comprehensive study [59]. In the current study, the subjects with SNP in (rs1050450) in *GPX1 gene (CC)* allele had lower concentrations of p-tGSH than the subjects with *(CT* + *TT) allele*. We also found that the concentrations of s-folate, p-tHcy and p-tGSH are not significantly different in subjects having *GSR (AA)* and *GSR (AG* + *GG)* alleles. These observations may suggest that the enzyme GSR maintains the redox GSH status in the cells, and do not have any impact on concentration of s-folate, p-tHcy and p-tGSH [35].

### Limitations

The results of this study should be interpreted with caution due to a relatively small sample size in a limited ethnic population receiving a low dose of folic acid supplementation over a short study period.

## Conclusion and future perspectives

The subjects with SNP (rs4880*) in SOD2 gene (CC allele), SNP* (rs1001179) in *CAT gene (CT* + *TT)* alleles and GST-T1 genotype have higher concentrations of s-folate and lower concentrations of p-tHcy than their counter parts. The study demonstrates that SNPs in genes coding antioxidant enzymes directly impact the concentrations of s-folate, p-tHcy and p-tGSH before and after folic acid supplementation. Clinical and biochemical studies may be initiated to investigate the impact of SNPs in antioxidant-enzyme genes on the concentrations of s-folate, p-tHcy and p-tGSH in patients with CVD and type 2 diabetes.

## Data Availability

No datasets were generated or analysed during the current study.
